# Do Intravenous Butaphosphan and Cyanocobalamin Combination Affect Insulin Resistance and Metabolic Profile of Dairy Goats During Their Transition Period?

**DOI:** 10.1002/vms3.70128

**Published:** 2024-11-22

**Authors:** Asghar Zare, Aliasghar Chalmeh, Mehrdad Pourjafar, Armin Amirian

**Affiliations:** ^1^ Department of Clinical Sciences School of Veterinary Medicine Shiraz University Shiraz Iran

**Keywords:** butaphosphan, cyanocobalamin, dairy goat, insulin resistance, transition period

## Abstract

**Background:**

Insulin resistance during early lactation in goats has been a topic of interest for researchers, as addressing this issue can significantly improve their metabolic health.

**Objectives:**

To investigate the potential of butaphosphan and cyanocobalamin in controlling insulin resistance, we conducted a study with the hypothesis that this combination may mitigate insulin resistance in dairy goats.

**Methods:**

Ten adult goats were divided equally into two groups: Ctrl and B+C. The Ctrl group received 6 mL of normal saline, while the second group was administered 6 mL of 10% butaphosphan and 0.005% cyanocobalamin on days 21, 20, 19, and 12, 11, 10, and 3, 2, 1 before parturition. On the 10th and 20th days after parturition, blood samples were gathered to analyze the levels of different metabolites and evaluate insulin resistance/sensitivity through an intravenous glucose tolerance test and surrogate indices. Body condition scores, milk production, and weight gain of the kids were also recorded during the study.

**Results:**

Although the B+C group showed slightly higher insulin responsiveness than the Ctrl group in the intravenous glucose tolerance test, but the difference was insignificant. Comparably, no significant differences were noticed in the remaining metabolic indicators amidst the Ctrl and B+C groups.

**Conclusions:**

The lack of substantial differences can be attributed to the limited sample size and the prescribed drug dosage. Further investigations with higher doses exceeding 6 mL are warranted to explore potential effects. Additionally, species‐specific differences in ruminants might exist, and caprine metabolism of the compound might differ from that of bovine and ovine. Consequently, we recommend conducting more studies in this field.

## Graphical abstract

This study was designed to answer whether butaphosphan and cyanocobalamin affect insulin resistance in transition dairy goats. This combination with prescribed amounts and frequency did not have a significant effect on the metabolic indices of dairy goats but enhanced their insulin responsiveness.

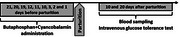

## 1 Introduction

Pregnancy, parturition, and lactation lead to various physiological changes in ruminants' homeostasis. Mismanagement of these changes can negatively impact their health and productivity (Agrawal et al. 2017). The critical period transitioning from a nonlactating pregnant to lactating nonpregnant state is known for significant metabolic needs and physiological alterations. The period above encompasses three weeks before to three weeks following parturition, is of utmost importance in properly managing dairy goats, as it plays a critical role in ensuring their overall health and performance. One of the challenges in this period is the increase in energy requirements and decrease in dry matter intake (DMI), which can result in or exacerbate metabolic disorders (Matthews 2016).

The rise in maternal energy requirements is primarily due to substantial fetal growth and lactogenesis towards the end of pregnancy. The ruminal capacity decreases due to fetal growth, leading to reduced DMI. These events cause negative energy balance (NEB), where energy intake decreases, and consumption increases (Esposito et al. 2014). As a result, the process of fat mobilization is initiated, thereby resulting in an escalation of nonesterified fatty acids (NEFA) and ketone bodies, including β‐hydroxybutyric acid (BHBA) in the bloodstream, ultimately leading to the occurrence of ketosis (Ai et al. 2018). In dairy goats, this condition is known as hyperketonemia or pregnancy toxemia, commonly occurring during transition period.

The NEB in dairy goats often leads to subclinical hyperketonemia, where animals do not show signs of clinical ketosis. However, they become vulnerable to other metabolic disorders around parturition, like mastitis, hypocalcemia, and insulin resistance (IR) (Simões and Gutiérrez 2017). IR, which refers to decreased sensitivity of body cells to insulin, is a significant metabolic disorder in ruminants during this time and can be a consequence or cause of NEB. Researchers have consistently sought solutions to reduce NEB and IR in ruminants. Furthermore, IR might be considered as a physiological adaptation in ruminants to optimally utilize food for supporting the pregnant uterus and lactating mammary gland (Zamuner et al. 2020). Nevertheless, while the uterus and mammary gland cells may have sufficient energy intake, other cells in the body lack energy, leading to metabolic disorders.

Therefore, it is essential to adopt management strategies to reduce the occurrence of IR. Researchers propose that providing energy during the transition period can effectively reduce NEB and related metabolic disorders like IR (Chibisa et al. 2008). Glucose supply is crucial for efficient metabolism in ruminants during NEB, but their glucose metabolism differs from other mammals. Ruminants primarily produce glucose through hepatic gluconeogenesis and from glucose precursors, mainly propionate (Chalmeh, Hajimohammadi, and Nazifi 2015). Consequently, using methods that provide glucose precursors and promote gluconeogenesis may effectively manage NEB, and subsequently impacting IR and reducing metabolic disorders.

Various nutritional and parenteral compounds have been utilized and recommended to address NEB and manage IR in ruminants. A notable combination involves the use of butaphosphan and cyanocobalamin. Previous studies have demonstrated the effectiveness of this combination in preventing and reducing IR during the transition period in dairy cows (Chalmeh et al. 2021) and sheep (Mohammadi Barimanloo et al. 2022). Interestingly, the combination of butaphosphan (as a source of organic phosphorus) and cyanocobalamin (as a gluconeogenesis stimulator) has been found to impact lactogenesis (Kreipe et al. 2011) positively and holds potential benefits in managing NEB for dairy cows and ewes alike (Pereira et al. 2013).

During parturition, cows experience a decrease in cyanocobalamin, a form of vitamin B12 (Rollin et al. 2010). It is hypothesized that administering vitamin B12 through injections could enhance gluconeogenesis by increasing the activity of methyl malonyl‐CoA mutase, an enzyme dependent on vitamin B12 in the tricarboxylic acid cycle (Girard and Matte 2006). Additionally, butaphosphan may enhance the efficiency of gluconeogenesis by phosphorylating the intermediates produced during the process, thereby facilitating the continuation of the tricarboxylic acid cycle (Berg, Tymoczko, and Stryer 2006). Some reports have indicated that animals receiving this combination experienced improved energy status (Rollin et al. 2010) and reduced IR (Chalmeh et al. 2021).

Based on the existing research and findings, the hypothesis emerges: Can intravenous administration of the combination of butaphosphan and cyanocobalamin to dairy goats at the end of pregnancy enhance their metabolic function after parturition? Another objective of this study is to assess the impact of this combination on the IR of dairy goats using dynamic and static tests. Additionally, by evaluating the metabolic biomarkers of goats in both the control and treatment groups, the effectiveness of this combination on the metabolism of dairy goats can be investigated. This study's findings may support utilizing the butaphosphan and cyanocobalamin combination to manage IR and other metabolic disorders in dairy goats.

## 2 Materials and Methods

### 2.1 Animal Selection

The study occurred on a Saanen dairy goat farm near Shiraz, Fars province, Iran. Before the study, the goats' clinical health was thoroughly examined to ensure their well‐being. Ten adult goats of the same age (2 years old) were chosen, all of whom underwent estrus synchronization and became pregnant simultaneously. The gestational age was estimated using ultrasonographic examinations and mating dates with the same buck. Only goats likely to have a single fetus based on ultrasonographic assessments were included in the study.

Furthermore, it is noteworthy that the chosen goats had demonstrated a track record of prosperous parturitions and were free from any reproductive complications. The goats were housed in an open‐shed barn, with unrestricted access to water and shade, and were provided with a well‐proportioned diet. Solely those goats with body condition scores ranging from 3.25 ± 0.25 were considered for inclusion in the study.

### 2.2 Grouping and Drug Administration

The goats were divided into Control (Ctrl) and butaphosphan + cyanocobalamin (B+C). The Ctrl group received normal saline, while the second group received a combination of butaphosphan 10% and cyanocobalamin 0.005% (Catosin; Erfandarou Pharmaceutical Company, Iran). The administration of drugs began 21 days before parturition, and subsequent injections were given as follows:

- In the 3rd week before parturition, drugs were administrated on days 21, 20, and 19 before parturition;
- In the 2nd week before parturition, drugs were administrated on days 12, 11, and 10 before parturition;
- In the last week before parturition, drugs were administrated on days 3, 2, and 1 before parturition.

The experimental group designated as Ctrl was administered a 6 mL of normal saline solution, whereas the experimental group B+C received a 6 mL of butaphosphan and cyanocobalamin combination.

### 2.3 Evaluating Serum and Metabolic Parameters

Blood samples were collected from the jugular vein of all goats on the 10th and 20th days after parturition. After clotting, the samples were centrifuged at 3000 rpm for 10 min, and the obtained sera were stored at –22°C until further analysis in the laboratory. The following serum indices were assessed: Glucose levels were measured using the enzymatic (glucose oxidase) colorimetric method provided by ZistChem; Tehran, Iran (sensitivity equal to 5 mg/dL; with intra‐assay and inter‐assay CV<10% and 11%, respectively). Insulin levels were analyzed using a goat‐specific insulin ELISA kit (Bioassay Technology Laboratory, China, Sensitivity equal to 0.058 mIU/L; the intra‐assay and inter‐assay CV were below 8% and 10%, respectively). NEFA and BHBA levels were measured using a colorimetric method provided by Randox, Ireland (The sensitivity for NEFA and BHBA was 0.072 mmol/L and 0.100 mmol/L, respectively; the intra‐assay and inter‐assay CV for NEFA were lesser than 7% and 11%, respectively, while for BHBA, it was lesser than 6%). Serum cortisol levels were determined using a competitive immunoenzymatic colorimetric method with the Diametra cortisol ELISA kit (Italy; sensitivity equal to 2.42 ng/mL, with intra‐assay and inter‐assay CV below 5.1% and 11%, respectively). Serum concentrations of total protein, albumin, aspartate aminotransferase (AST), and gamma‐glutamyl transferase (GGT) were analyzed using kits manufactured by Pars Azmoon CO, Iran. Concentrations of triglyceride (TG), cholesterol, alanine transaminase (ALT), and total and direct bilirubin were analyzed using Paadco CO kits; Iran. The Biorexfars CO kits from Iran were used to measure the serum levels of both high‐density lipoprotein (HDL) and low‐density lipoprotein (LDL). Serum globulin levels were calculated by subtracting albumin levels from total protein. Indirect bilirubin levels were determined by subtracting direct bilirubin levels from total bilirubin values. Serum concentrations of very low‐density lipoprotein (VLDL) were determined using the Friedewald, Levy, and Fredrickson (1972) method, which estimates the VLDL levels as one‐fifth of the concentration of TG.

### 2.4 Evaluating Insulin Resistance

#### 2.4.1 Dynamic Test: Intravenous Glucose Tolerance Test (ivGTT)

The ivGTT was performed on two occasions, precisely 10 and 20 days after parturition in Ctrl and B+C groups. The testing procedure followed the recommendations provided by De Koster and Opsomer (2013). Before each test, the animals underwent a 12‐h fasting period with continuous access to water. Approximately an hour before the ivGTT, the animals' weights were recorded. A 16 G 5.1 cm intravenous catheter was inserted carefully into the jugular vein and secured on the skin using a single subcutaneous suture loop. The first blood sample was collected after approximately 30 min when the goats were calm to minimize the stress caused by catheterization.

Blood samples were taken to establish baseline insulin and glucose concentrations before administering glucose. Glucose was intravenously administered as a 50% solution (Zoopha Parnian Pars Company, Iran) at 0.5 g/kg BW. The total glucose volume was infused using a syringe over 1 min. The catheter was flushed with 15 mL of 0.9% NaCl solution (Shahid Ghazi Pharmaceutical CO, Tabriz, Iran) into the jugular vein to prevent impregnation of the blood samples with the infused glucose.

A total of 10 blood samples were collected at intervals of 7 min during the 63 min following glucose administration. These samples were collected in 10‐mL plain tubes, stored within 2 h at room temperature, and centrifuged subsequently (3000 rpm for 10 min). The resulting sera were maximally 10 days stored at –22°C until analyses.

Subsequently, glucose and insulin concentrations were analyzed in all specimens to calculate various ivGTT indices. Glucose and insulin concentrations were measured every 7 min after glucose administration and the following indices were computed: basal glucose concentration before glucose administration (G0), peak glucose concentration at 7 min after intravenous glucose injection (GMAX), glucose half‐life time (GHLT) at which GMAX reached half its value, glucose area under the curve (GAUC) calculated using the trapezoidal method, and glucose concentration at minute 63 (G63). G63:G0 was determined by dividing the glucose concentration at minute 63 by the glucose concentration at minute 0.

This study assessed several distinct insulin responses, including the basal insulin concentration before glucose injection (I0), the peak insulin concentration between 7 and 21 min after intravenous glucose injection (IMAX), the insulin concentration at minute 63 (I63), and the insulin area under the curve (IAUC) determined using the trapezoidal methodology. I63:I0 was calculated by dividing the insulin concentration at minute 63 by the insulin concentration at minute 0. According to De De Koster and Opsomer's (2013) guidelines, GAUC, GHLT, G63:G0, IAUC, and I63:I0 are considered appropriate parameters for evaluating insulin sensitivity or responses to ivGTT among the studied groups.

The glucose elimination rate (GER) and insulin elimination rate (IER) during the ivGTT were determined based on the formula proposed by Pires, Souza, and Grummer (2007).

#### 2.4.2 Nondynamic Tests (Surrogate Indices)

Various indices have been proposed as nondynamic tests to estimate IR levels (De Koster and Opsomer 2013). The primary objective of these tests is to predict IR in peripheral tissues based on fasting blood samples. The following formulas have been employed to achieve this, with all factors derived from the initial blood sample before glucose infusion:

$$\begin{array}{c} \mathrm{HOMA}-\mathrm{IR}=\left[\mathrm{glucose}\left(\mathrm{mmol}/\mathrm{mL}\right)\times \mathrm{insulin}\left(\mu U/\mathrm{mL}\right)\right]/22.5\\ \mathrm{QUICKI}=1/\left[\mathrm{logglucose}\left(\mathrm{mg}/\mathrm{dL}\right)+\mathrm{loginsulin}\left(\mu U/\mathrm{mL}\right)\right]\\ \mathrm{RQUICKI}=1/\left[\mathrm{logglucose}\left(\mathrm{mg}/\mathrm{dL}\right)+\mathrm{loginsulin}\left(\mu U/\mathrm{mL}\right)+\mathrm{logNEFA}\left(\mathrm{mmol}/L\right)\right]\\ \mathrm{RQUICK}I_{\mathrm{BHBA}}=1/\left[\mathrm{logglucose}\left(\mathrm{mg}/\mathrm{dL}\right)+\mathrm{loginsulin}\left(\mu U/\mathrm{mL}\right)+\mathrm{logNEFA}\left(\mathrm{mmol}/L\right)+\mathrm{logBHBA}\left(\mathrm{mmol}/L\right)\right] \end{array}$$

### 2.5 Evaluating Body Condition Scores

Goats were assessed through a visual and tactile examination, utilizing a 5‐point scale with intervals of 0.25 from 20 days before parturition to 60 days postparturition. Within this system, a score of 1 signifies a state of pronounced emaciation, whereas a score of 5 denotes marked obesity (Kenyon, Maloney, and Blache 2014).

### 2.6 Milk Production

Milk production (kg/day) of all the goats under study was assessed every 10 days until 60 days after parturition.

### 2.7 Weight of the Kids

The kids' weight was recorded every 10 days from birth until they reached 60 days old.

### 2.8 Statistical Analyses

Data are expressed as mean and standard error (SE) and, with a specific focus on the standard error of the mean (SEM), which was also displayed in some tables. To evaluate group differences at each study period, the two independent samples *t*‐test was employed, whereas the paired samples *t*‐test was utilized to assess differences within the same group between days 10 and 20 after parturition. The effects of treatment, time, and interaction on the measured variable concentrations were investigated using Repeated Measures ANOVA. The software employed for statistical analysis in the current investigation consisted of SPSS (SPSS for Windows, version 22, SPSS Inc, Chicago, IL, USA). A mean difference was deemed significant at *p* < 0.05 to ascertain statistical significance.

## 3 Results

Table 1 presents the basal levels of the studied parameters (mean ± SE) for the Ctrl and treatment groups 10 and 20 days after parturition. According to the data, the serum concentration of insulin and glucose was higher in the B+C group compared to the Ctrl group. Insulin levels decreased, while glucose concentrations increased in both groups during the study. Additionally, the B+C group exhibited lower serum concentrations of NEFA, cortisol, lipid profile, and protein profile than the Ctrl group in both study periods. Cortisol concentration on day 20 after parturition was lower in both groups compared to day 10, and this difference was significant in the treatment group. The lipid and protein profile values increased and decreased, respectively, throughout the study. On the 10th day after parturition, total, direct, and indirect bilirubin levels were lower in the B+C group than in Ctrl, but this trend reversed on the 20th day after parturition. The changes in bilirubin levels did not follow a specific pattern during the study. The serum levels of BHBA and hepatic enzymes were not different significantly across various groups and during different study phases. Furthermore, alterations in their levels from day 10 to day 20 postparturition lacked any distinct pattern.

**TABLE 1:** The basal levels of studied parameters (mean ± SE) of Ctrl and treatment groups at 10 and 20 days after parturition.

Parameters	Groups	10 days AP	20 days AP	*p* value^a^
Insulin (mIU/L)	Ctrl	6.11 ± 0.12	3.21 ± 0.68	0.020^*^
	B+C	9.55 ± 4.01	4.88 ± 0.37	0.314
Glucose (mg/dL)	Ctrl	48.1 ± 4.93	48.7 ± 4.59	0.807
	B+C	34.54 ± 6.95	62.5 ± 20.18	0.260
BHBA (mmol/L)	Ctrl	0.10 ± 0.004	0.10 ± 0.003	0.217
	B+C	0.11 ± 0.006	0.10 ± 0.001	0.309
NEFA (mmol/L)	Ctrl	0.5 ± 0.1	0.47±0.1	0.401
	B+C	0.45 ± 0.04	0.45 ± 0.2	0.981
Cortisol (ng/ml)	Ctrl	0.37 ± 0.15	0.35 ± 0.19	0.932
	B+C	0.18 ± 0.04	0.05 ± 0.03	0.041^*^
TG (mg/dL)	Ctrl	16.48 ± 1.65	12.5 ± 1.05	0.057
	B+C	12.98 ± 1.24	15.92 ± 1.96	0.373
Cholesterol (mg/dL)	Ctrl	74.5 ± 4.22	76.3 ± 4.73	0.547
	B+C	71.2 ± 5.84	75.1 ± 7.63	0.687
HDL (mg/dL)	Ctrl	38.3 ± 1.96	38.4 ± 2.46	0.962
	B+C	36 ± 2.43	38.1 ± 2.54	0.632
LDL (mg/dL)	Ctrl	32.92 ± 2.4	35.38 ± 2.9	0.329
	B+C	32.6 ± 3.59	33.84 ± 5.72	0.841
VLDL (mg/dL)	Ctrl	3.29 ± 0.33	2.5 ± 0.21	0.057
	B+C	2.59 ± 0.24	3.18 ± 0.39	0.373
Total protein (g/dL)	Ctrl	7.9 ± 0.29	7.58 ± 0.28	0.173
	B+C	7.42 ± 0.12	7.24 ± 0.26	0.468
Albumin (g/dL)	Ctrl	4.05 ± 0.09	3.85 ± 0.10	0.025^*^
	B+C	3.9 ± 0.11	3.75 ± 0.16	0.389
Globulin (g/dL)	Ctrl	3.84 ± 0.25	3.72 ± 0.21	0.457
	B+C	3.51 ± 0.15	3.48 ± 0.16	0.749
Total bilirubin (mg/dL)	Ctrl	0.30 ± 0.05	0.25 ± 0.02	0.470
	B+C	0.25 ± 0.02	0.27 ± 0.01	0.683
Direct bilirubin (mg/dL)	Ctrl	0.17 ± 0.02	0.15 ± 0.003	0.371
	B+C	0.14 ± 0.01	0.13 ± 0.01	0.916
Indirect bilirubin (mg/dL)	Ctrl	0.13 ± 0.03	0.10 ± 0.02	0.578
	B+C	0.11 ± 0.02	0.13 ± 0.01	0.726
AST (U/L)	Ctrl	87 ± 3.67	87.9 ± 2.93	0.667
	B+C	102.1 ± 10.51	101.1 ± 9.53	0.789
ALT (U/L)	Ctrl	15.42 ± 1.47	15.82 ± 1.06	0.553
	B+C	13.58 ± 0.85	14.8 ± 0.97	0.356
GGT (U/L)	Ctrl	37.5 ± 3.4	38 ± 3.51	0.746
	B+C	36 ± 1.62	40.1 ± 4.29	0.268

AP: after parturition; Ctrl: control; B+C: butaphosphan + cyanocobalamin; BHBA: beta‐hydroxybutyric acid; NEFA: nonesterified fatty acids; TG: triglycerides; HDL: high‐density lipoprotein; LDL: low‐density lipoprotein; VLDL: very low‐density lipoprotein; AST: aspartate aminotransferase; ALT: alanine transaminase; GGT: gamma‐glutamyl transferase.

^a^ *p* Values presented in this column represents statistical differences between same parameters in similar groups at different days and stars in this column indicate significant differences (*p* < 0.05).

**FIGURE 1:**
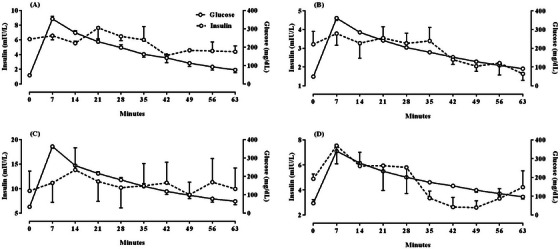
Alterations of insulin and glucose following intravenous glucose tolerance test (ivGTT) of dairy goats at 10 and 20 days after their parturition. Ctrl group received 6 mL intravenous normal saline and the treatment group received 6 mL 10% butaphosphan and 0.005% cyanocobalamin combination on days 21, 20, 19, and 12, 11, 10, and 3, 2, 1 before parturition. A: Ctrl group at 10 days after parturition; B: Ctrl group at 20 days after parturition; C: Treatment group at 10 days after parturition; D: Treatment group at 20 days after parturition.

Table 2 displays the trend of changes in glucose and insulin following intravenous injection of 50% dextrose in the ivGTT during different study periods. The indices under investigation showed no significant differences between the two study groups during the same time intervals. However, the trend of glucose changes on days 10 and 20 after parturition was found to be significant. Furthermore, the pattern of insulin changes following the ivGTT on the 10th day after parturition was insignificant but became noteworthy on the 20th day after. Drawing from the outcomes presented in Table 2, we generated Figure 1, which illustrates the marked elevation in glucose concentration across all groups and study periods, particularly in the seventh minute following the intravenous administration of 50% dextrose. This conspicuous finding is indicative of a significant metabolic response. Simultaneously, the pattern of insulin changes gradually increased in all groups, and subsequently, both glucose and insulin levels started to decrease. The trend of glucose changes in all periods and groups was consistent and significant, aligning with the findings presented in Table 2. However, the changes of insulin in different groups and periods varied, with significance observed on the 20th day after parturition while insignificant on the 10th day.

**TABLE 2:** Alterations of glucose and insulin (mean ± SEM) following ivGTT in different groups at 10 and 20 days after parturition.

			Minutes after glucose infusion (mean)										SEM	*p* value^a^		
Parameters	Periods	Groups	0	7	14	21	28	35	42	49	56	63		Treatment	Time	Treatment×Time
Glucose (mg/dL)	10 days AP	Ctrl	48.1	354.4	278.8	230	198.2	161.8	141.9	113.7	93.6	76.8	14.3	0.399	<0.001*	0.948
		B+C	34.54	362.8	258.6	215.6	180.8	146.6	118.5	97.11	78.2	64.5	13.8			
	20 days AP	Ctrl	48.7	361	285	241.8	204.4	178.8	152.4	129.2	108.7	91.2	6.54	0.779	<0.001*	0.687
		B+C	62.5	340.6	276.6	232.6	200.6	173.8	154.8	131.8	113.4	96.5	9.31			
Insulin (mIU/L)	10 days AP	Ctrl	6.11	6.56	5.56	7.63	6.49	6	3.88	4.58	4.49	4.39	0.78	0.320	0.139	0.306
		B+C	9.55	11.16	13.82	11.46	10.2	10.56	11.2	8.76	11.32	9.95	4.09			
	20 days AP	Ctrl	3.21	3.78	3.26	3.54	3.26	3.38	2.39	2.04	2.21	1.63	0.54	0.102	<0.001*	0.048*
		B+C	4.88	7.54	5.91	5.94	5.79	3.34	2.62	2.59	3.31	4.21	1.1			

AP: after parturition.

^a^ *p* Values reveal the effects of treatment, time and treatment × time during ivGTT at each study day and stars indicate significant effects (*p* < 0.05).

Table 3 presents the investigated indicators used to evaluate the degree of IR. The data exhibited no dissimilarity of statistical significance between the reactions of the animals under study toward the ivGTT on same days. Furthermore, no variation was observed between these indices on the 10th and 20th days following parturition.

**TABLE 3:** Computational indices for evaluating insulin resistance following the results of ivGTT in Ctrl and treatment groups at different study periods.

Indices	Groups	Periods (mean)		*p* value
		10 days AP	20 days AP	
G0 (mg/dL)	Ctrl	48.1 ± 4.93	48.7 ± 4.59	0.807
	B+C	34.54 ± 6.95	62.5 ± 20.18	0.990
GMAX (mg/dL)	Ctrl	354.4 ± 14.1	361 ± 12.72	0.543
	B+C	362.8 ± 9.16	340.6 ± 8.4	0.354
GHLT (Min)	Ctrl	29.4 ± 5.6	30.8 ± 2.8	0.704
	B+C	23.8 ± 3.56	35 ± 3.13	0.051
GAUC (mg/dL/63 min)	Ctrl	11443.95 ± 728.51	12118.75 ± 180.06	0.332
	B+C	10701.16 ± 678.07	11925.9 ± 349.08	0.097
G63 (mg/dL)	Ctrl	76.8 ± 15.12	91.2 ± 8.96	0.235
	B+C	64.5 ± 18.97	96.5 ± 10.64	0.137
G63:G0	Ctrl	1.66 ± 0.33	1.93 ± 0.23	0.211
	B+C	2.9 ± 1.61	2.06 ± 0.46	0.276
GER (%/min)	Ctrl	0.77 ± 0.1	0.65 ± 0.04	0.263
	B+C	1.06 ± 0.15	0.60 ± 0.05	.441
I0 (mIU/L)	Ctrl	6.11 ± 0.12	3.21 ± 0.68	0.221
	B+C	9.55 ± 4.01	4.88 ± 0.37	0.911
IMAX (mIU/L)	Ctrl	8.70 ± 1.07	3.93 ± 0.7	0.585
	B+C	14.24 ± 4.36	7.55 ± 1.54	0.497
IAUC (mIU/L/63 min)	Ctrl	353.39 ± 33.94	184.27 ± 31.61	0.014*
	B+C	687.86 ± 249.1	291.41 ± 63.90	0.150
I63 (mIU/L)	Ctrl	4.39 ± 0.79	1.64 ± 0.33	0.809
	B+C	9.95 ± 4.27	4.21 ± 1.3	0.313
I63:I0	Ctrl	0.71 ± 0.12	0.56 ± 0.09	0.760
	B+C	0.96 ± 0.09	0.83 ± 0.23	0.866
IER (%/min)	Ctrl	8.89 ± 1.12	15.92 ± 7.58	0.227
	B+C	15.01 ± 9.19	12.15 ± 3.09	0.804

AP: after parturition; Ctrl: control; B+C: butaphosphan + cyanocobalamin; G0: the basal glucose concentration before glucose administration; GMAX: peak glucose concentration assessed 7 min after intravenous glucose injection; GHLT: glucose half‐life; GAUC: glucose area under the curve; H63: glucose concentration at minute 63; G63:G0: calculated by dividing glucose at minute 63 by glucose at minute 0; GER: glucose elimination rate; I0: the basal insulin concentration before glucose injection; IMAX: peak insulin concentration between 7 and 21 min after intravenous glucose injection; IAUC: insulin area under the curve; I63: insulin concentration at the end of the ivGTT; I63:I0: calculated by dividing insulin at minute 63 by insulin at minute 0; IER: insulin elimination rate.

^a^ *p* Values presented in this column represents statistical differences between same parameters in similar groups at different days and stars in this column indicate significant differences (*p* < 0.05).

The trends of changes in BCS and milk production of goats, and the weight of their kids are displayed in Table 4. Based on the findings, there was no statistically significant difference between the values of these indicators in the Ctrl and B+C groups on the same days. Furthermore, the trend of changes in BCS and milk production of goats during the study was insignificant, but kids' weight significantly increased with age.

**TABLE 4:** Alterations of BCS and milk yield of studied goats, and their kids’ weight (mean ± SEM) during the study period at different groups.

		Days										*p* value^a^		
Parameters	Groups	20 BP	10 BP	*p*	10 AP	20 AP	30 AP	40 AP	50 AP	60 AP	SEM	Treatment	Time	Treatment × Time
BCS	Ctrl	3.35	3.35	3.35	3.15^a^	3	3	2.95	2.95	3.1	0.11	0.134	0.063	0.454
	B+C	2.95	2.95	2.95	2.7^b^	2.7	2.75	2.8	2.8	2.95	0.14			
Milk yield (kg)	Ctrl	—	—	1.42	2.2	2.22	2.29	2.24	1.99	2.08	0.14	0.590	0.064	0.145
	B+C	—	—	1.41	2.1	2.1	2.2	2.54	2.68	2.64	0.31			
Kids’ weight (kg)	Ctrl	—	—	3.44	4.26	4.8	5.38	6.14	7.2	7.94	0.36	0.476	0.005^*^	0.043^*^
	B+C	—	—	3.22	4.24	4.97	5.6	6.4	7.6	8.9	0.20			

BCS: body condition score; AP: after parturition; CTRL: control; B+C: B+C: butaphosphan + cyanocobalamin.

^a,d^ Different letters indicate significant difference between different groups at same day (*p* < 0.05).

^a^ *p* Values reveal the effects of treatment, time and treatment × time during the study period and stars indicate significant effects (*p* < 0.05).

Table 5 is derived from the results of static tests used to assess insulin resistance/sensitivity (HOMA‐IR, QUICKI, RQUICKI, and RQUICKI_BHBA_). Based on the obtained findings, it can be inferred that no significant statistical difference was detected among the distinct cohorts on the respective days. Moreover, the fluctuating trends observed in the outcomes of these assessments during the study were deemed insignificant.

**TABLE 5:** Computational indices for static evaluation of insulin resistance/sensitivity of Ctrl and treatment groups at different study days based on the parameters before intravenous dextrose 50% infusion.

Indices	Groups	Periods		*p* value^a^
		10 days AP	20 days AP	
HOMA‐IR	Ctrl	0.73 ± 0.07	0.39 ± 0.1	0.018^*^
	B+C	0.61 ± 0.22	0.8 ± 0.31	0.661
QUICKI	Ctrl	0.4 ± 0.01	0.47 ± 0.03	0.076
	B+C	0.45 ± 0.03	0.41 ± 0.02	0.451
RQUICKI	Ctrl	0.48 ± 0.03	0.6 ± 0.07	0.060
	B+C	0.55 ± 0.07	0.64 ± 0.11	0.605
RQUICKI_BHBA_	Ctrl	1 ± 0.17	2.13 ± 0.89	0.198
	B+C	1.7 ± 0.8	−2 ± 3.19	0.309

AP: after parturition; Ctrl: control; B+C: butaphosphan + cyanocobalamin; HOMA‐IR: homeostasis model assessment‐insulin resistance; QUICKI: quantitative insulin sensitivity check index; RQUICKI: revised quantitative insulin sensitivity check index; RQUICKI_BHBA_: revised quantitative insulin sensitivity check index‐beta hydroxy butyric acid.

^a^ *p* Values presented in this column represents statistical differences between same indices in similar groups at different days and star in this column indicates significant difference (*p* < 0.05).

## 4 Discussion

The main aim of this study was to investigate the effects of administering butaphosphan and cyanocobalamin intravenously to dairy goats during the late pregnancy period, as well as to assess their IR through dynamic and static evaluations after parturition. The findings indicated that administering this compound before parturition did not significantly impact the goats' metabolic indicators and insulin responsiveness postparturition. Although the B+C group showed a slightly higher baseline insulin concentration on the 10th and 20th days after giving birth than the control group, this difference was not considered statistically significant (Table 1; *p* > 0.05). However, the intravenous injection of 50% dextrose elicited a clear and substantial response in all goats. This response was evident in the simultaneous increase in blood glucose and insulin concentrations within the seventh minute after the injection (Table 2 and Figure 1; *p* < 0.05).

Table 3 presents the computational indices for evaluating insulin responsiveness following the ivGTT, which showed no statistically significant difference between groups and days. Nonetheless, specific indicators, such as GAUC, decreased within the treatment cohort relative to the control cohort, implying a heightened susceptibility of goats within the B+C group to insulin, thus facilitating the more efficient clearance of glucose from the bloodstream. Additionally, it was observed that there existed no discernible differences in insulin indicators post the administration of the ivGTT, as delineated in Table 3. However, particular indices, encompassing I0, IMAX, IAUC, I63, and I63:I0, manifested an elevation within the B+C group compared to the control group, signifying an augmented insulin responsiveness within the B+C goats.

Although no statistically significant disparity was observed in the metabolic indices and insulin responsiveness across the various groups and periods, an elevation in the insulin response levels was detected in the B+C group as opposed to the control goats. The observed outcome could be attributed to the combination of butaphosphan and cyanocobalamin. It should be noted that this study had limitations regarding the number of animals used within the specified timeframe. Further studies with more groups or varying doses may yield more definitive results and require more investigation.

Previous research by other scholars has highlighted the presence of IR in goats during the early lactation period. For example, using an intravenous insulin challenge test Schmidely et al. (1993), explored the impact of milk production and physiological condition on IR in goats. They concluded that goats producing more milk during early lactation exhibited higher IR than other goats, possibly reducing glucose utilization in extramammary tissues. Similarly, Debras et al. (1989) investigated insulin sensitivity and responsiveness in Alpine dairy goats at different stages of lactation and the dry period, utilizing the euglycemic‐hyperinsulinemic clamp technique. Their study revealed a notable decline in glucose utilization response during the initial stages of lactation and a remarkable increase during the dry period. The results of the study suggest that there is a decrease in insulin sensitivity in peripheral tissues during early lactation. This decrease may be a protective mechanism to conserve gluconeogenic reserves and substrates. Moreover, the endocrinological and metabolic reactions to glucose and insulin injections in Saanen dairy goats during early lactation were examined by Zamuner et al. (2020), with differentiation between high and low‐milk‐producing groups. They employed ivGTT and insulin tolerance tests (ITT). They observed that goats with lower milk production displayed a significantly greater pancreatic response in insulin secretion than those with higher milk production. In contrast, no differences were observed in tissue responses to glucose and insulin injection between the groups. Based on this, they suggested that milk production directly impacts insulin responsiveness in dairy goats during early lactation.

The present study investigated insulin resistance/sensitivity in Saanen dairy goats using static and surrogate indices (Table 5). The computational indices yielded outcomes concordant with the discoveries of the ivGTT. The data exposed no noteworthy dissimilarity between the Ctrl group and the cohort that was given a mixture of butaphosphan and cyanocobalamin (B+C). In a study by Liu et al. (2021), surrogate indices were employed to assess IR in healthy Guanzhong goats and those with subclinical hyperketonemia. Weekly blood samples were obtained from three weeks pre‐partum to three weeks postpartum, and subsequently, the HOMA‐IR, QUICKI, RQUICKI, and RQUICKI_BHBA_ were computed. The study found that goats with subclinical hyperketonemia had significantly higher insulin levels compared to healthy goats in the weeks following parturition. Furthermore, HOMA‐IR was significantly higher in goats with subclinical hyperketonemia during the first week after parturition, while QUICKI, RQUICKI, and RQUICKIBHBA were substantially lower. These findings were attributed to the hemostatic reactions of dairy goats during early lactation as they try to maintain adequate blood glucose concentrations. Zamuner et al. (2021) conducted a study examining the utility of the dynamic ivGTT and static tests, namely HOMA‐IR, QUICKI, and RQUICKI, in assessing IR and their interrelationship in Saanen dairy goats during the early lactation phase. The results of the investigation indicated that there was no significant association between the dynamic and static test outcomes. The authors, therefore, concluded that the static indices are not appropriate for evaluating IR in the early lactation stage.

The current study's findings suggest that the intravenous injection of a combination of butaphosphan and cyanocobalamin did not significantly impact IR and metabolic indices in Saanen dairy goats. However, it is worth considering that higher doses or more frequent injections might yield different effects. The basis for this study's hypothesis stemmed from previous research on cows and ewes during the transition period, which revealed significant metabolic effects of intravenous infusion of butaphosphan and cyanocobalamin. These effects included reduced IR in cows and improved indicators related to ketosis and pregnancy toxemia in ewes (Chalmeh et al. 2021; Mohammadi Barimanloo et al. 2022). Despite these effects observed in other ruminants, our study did not find a statistically significant difference in dairy goats, possibly due to species‐specific differences.

Dairy goats, particularly those with twin pregnancies, face NEB during the transition and rely on fat stores to compensate. A key issue during this period is the reduced responsiveness and sensitivity to insulin. However, the glucose requirement increases significantly during this time (Castagnino et al. 2015). IR is a normal adaptation in healthy pregnant ruminants during late pregnancy and early lactation. These adjustments are necessary to ensure an adequate supply of glucose to the developing offspring and support their growth before and after birth. Reducing insulin sensitivity towards the end of pregnancy provides sufficient glucose transfer from the mother to the fetus, preventing hypoglycemia and ensuring survival. Researchers have studied metabolic and endocrine responses in dairy cows and found the highest level of IR during the transition period, highlighting it as a multifactorial issue with an unknown exact cause (Chalmeh et al. 2021).

Various supplements have been extensively studied to explore their impact on insulin sensitivity or resistance (Chalmeh et al. 2020; Chalmeh et al. 2021; Mohammadi Barimanloo et al. 2022). Among these, the combination of butaphosphan and cyanocobalamin has garnered attention. Recent research indicates that this combination enhances hepatic mRNA expression of LXRα, a crucial regulator in lipid metabolism that stimulates the citric acid cycle (Nuber, van Dorland, and Bruckmaier 2016). During early lactation in ruminants after giving birth, the liver undergoes significant adaptations, and many experience hepatic lipidosis to varying degrees (Hayirli 2006). Enhancing liver health and energy processing capacity could influence NEB in early lactation (De Koster and Opsomer 2013). Butaphosphan, an organophosphorus compound, plays a significant role in hepatic carbohydrate metabolism, where phosphorylation of all intermediates in the gluconeogenic pathway is necessary. Phosphorus availability is crucial in regulating the rates of gluconeogenesis and glycolysis (Berg, Tymoczko, and Stryer 2006). Some researchers have hypothesized that insufficient cyanocobalamin supply could be responsible for the lack of lactation response to folic acid supplementation in dairy cows (Girard et al. 2005). Administering intramuscular injections of cyanocobalamin to cows in early lactation has increased total milk solids and fat production, possibly due to the numerical increase in milk production. Additionally, cyanocobalamin decreases milk urea concentration, indicating a reduction in amino acid catabolism (Girard and Matte 2005). Cobalt is an essential element for cyanocobalamin synthesis in the rumen, located at the center of the cyanocobalamin chorine ring (McDowell 2000). Studies have demonstrated that higher cobalt intake positively affects cyanocobalamin levels (Kincaid et al. 2003; Stangl et al. 2000), and dietary cobalt content promotes rumen cyanocobalamin synthesis (Tiffany, Fellner, and Spears 2006).

Gordon et al. (2017) investigated the impact of butaphosphan and cyanocobalamin on ketosis occurrence in early lactation dairy cows and found promising results, suggesting that this compound could be a beneficial treatment for ketosis in dairy cows. In another study involving ewes, researchers examined the concurrent use of butaphosphan and cyanocobalamin to prevent pregnancy toxemia during the peripartum period (Temizel et al. 2015). Although the levels of BHBA and NEFA were notably lower in the group receiving the combination, the difference was not statistically significant. Combining butaphosphan and cyanocobalamin might offer an alternative treatment to prevent pregnancy toxemia. Tabeleão et al. (2017) studied the effect of combining butaphosphan and cyanocobalamin on postpartum dairy cows' glucose metabolism. Glucose tolerance tests and insulin challenges were performed on specific days after calving. The investigators observed an absence of noteworthy dissimilarity in glucose metabolism among the cohorts. They concluded that the combined use of butaphosphan and cyanocobalamin positively influenced glucose metabolism adaptation in dairy cows during early lactation.

Rollin et al. (2010) investigated the effect of injecting butaphosphan and cyanocobalamin on the day of parturition and the following day on subclinical ketosis prevalence in early postpartum dairy cows. Their results suggested that this compound may reduce the incidence of subclinical ketosis during the first week after calving in mature dairy cows. Pereira et al. (2013) examined the effect of butaphosphan and cyanocobalamin supplementation on plasma metabolites in postpartum dairy cows. Increasing the doses of butaphosphan and cyanocobalamin resulted in a linear decrease in the plasma concentration of NEFA and BHBA. Additionally, another study investigated the effects of butaphosphan alone or in combination with cyanocobalamin on the metabolism of cows with subclinical ketosis in early lactation (Nuber, van Dorland, and Bruckmaier 2016). The results indicated that administering cyanocobalamin and butaphosphan to early lactating ketotic dairy cows affected lipid metabolism by influencing plasma metabolites, likely through modified liver activity.

Previous research on sheep and cattle did not reveal any significant changes in cortisol levels when investigating the impact of butaphosphan and cyanocobalamin on this hormone. However, it was observed that the combination of these substances reduced cortisol secretion in heifers, although the exact mechanism remains unclear (Hänsel et al. 1992). In the present study, we found no significant differences in cortisol levels between the Ctrl and B+C groups (Table 1; *p* > 0.05). Vitamin B12 has been shown to influence the function of enzymes involved in lipid metabolism, suggesting that a deficiency in this vitamin could alter lipid metabolism. However, our study and other research found no significant effects on cholesterol levels or lipomobilization indices (NEFA and BHBA) in response to butaphosphan and cyanocobalamin supplementation (Nuber, van Dorland, and Bruckmaier 2016; Rollin et al. 2010; Tabeleão et al. 2017). Furthermore, we evaluated liver health by assessing various biomarkers, including total protein, albumin, globulin, total, direct, and indirect bilirubin, AST, ALT, and GGT (Table 1). However, it was noted that there was an absence of significant differences between the groups, thus indicating that the compound had no discernible effect on hepatic functionality.

As discussed the beneficial impact of butaphosphan and cyanocobalamin on the metabolic processes and negative energy balance of dairy cows and ewes, we conducted a practical investigation into the effect of this compound on Saanen dairy goats, a key hypothesis in our research. As a result, we meticulously assessed a wide range of metabolic indicators in this study, with the outcomes presented in Table 1. Based on the presented results in Table 1, it seems reasonable to attribute these findings to species and breed differences in metabolic function. Moreover, the results obtained indicate that the administration of butaphosphan and cyanocobalamin, as carried out in this study, yields the results outlined in Table 1. It's important to note that studies conducted on goats with a different design from ours may produce different results.

The findings of our current investigation demonstrated that the administration of 6 mL of 10% butaphosphan and 0.005% cyanocobalamin during the prepartum phase did not result in any noteworthy dissimilarities in BCS of goats or the weight of their kids; Moreover, it did not significantly affect their milk production (Table 4). Interestingly, this finding contrasts with our previous studies on sheep, where lambs from mothers who received the same compound had significantly higher weights (Mohammadi Barimanloo et al. 2022). In that study, we speculated that the increased metabolism in ewes may have contributed to reduced NEB in the treatment group, leading to the observed weight gain in their offspring.

In the present study, we raised the hypothesis of whether the administration of the combination of butaphosphan and cyanocobalamin can improve insulin resistance in pregnant goats after parturition. This hypothesis is based on the study records of the authors on the effects of this combination in dairy cows and pregnant ewes, as well as other studies in this field. Our previous studies on dairy cows and sheep showed that this compound can change the insulin resistance in these animals. However, our study on goats, as a ruminant, yielded different results. It is worth noting that species and breed differences should be taken into account. The study used Saanen goats, while we used Holstein cows (Chalmeh et al. 2021) and Afshari sheep (Mohammadi Barimanloo et al. 2022) in our previous studies. It is suggested that using different breeds may yield different results, but further research is needed. It is also important to consider the treatment protocol, drug doses, and specific breed of goats used in this study when interpreting the results. These results can guide future studies and researchers can design their studies differently to achieve other results. It is possible that the combination of butaphosphan and cyanocobalamin may not have the same effects in goats as it does in cows and sheep.

## 5 Conclusion

The results of the current study suggested that administering a 6 mL solution containing 10% butaphosphan and 0.005% cyanocobalamin intravenously to Saanen dairy goats at their late pregnancy had an impact on various IR‐related measures, insulin sensitivity, and insulin responsiveness during early lactation. Although the goats receiving this compound showed improved insulin responsiveness, the differences between the treatment and control groups did not reach statistical significance. These nonsignificant differences may be attributed to the limited number of animals in each group and the prescribed dosage, which was determined based on the manufacturer's guidelines and our previous research. It is essential to acknowledge that the lack of significant differences might also be influenced by potential alternative effects with doses exceeding 6 mL, which calls for further investigation through further studies. Additionally, differences in metabolic processes among ruminant species, such as cows and sheep, could contribute to the absence of significant differences in goats. Therefore, conducting more research in this area is strongly recommended for a comprehensive understanding.

## Author Contributions

**Asghar Zare**: Investigation, project administration, writing—review & editing. **Aliasghar Chalmeh**: Conceptualization, methodology, formal analysis, investigation, resources, data curation, writing original draft, writing—review & editing, supervision, project administration. **Mehrdad Pourjafar**: Validation, resources, writing—review & editing, supervision, project administration. **Armin Amirian**: Software, Formal analysis, data curation, writing—review & editing, project administration.

## Ethical Statement

The present experimental study adhered to the ethical guidelines for laboratory animal research set forth by the Iranian laboratory animal ethics framework. The study was closely monitored by the Iranian Society for the Prevention of Cruelty to Animals and the Shiraz University Research Council (IACUC no: 4687/63).

## Conflicts of Interest

There is no conflict of interest.

## Data Availability

The datasets generated and analyzed during the current study are available from the corresponding author upon reasonable request.
